# Designing and Creating a Synthetic Omega Oxidation Pathway in *Saccharomyces cerevisiae* Enables Production of Medium-Chain α, ω-Dicarboxylic Acids

**DOI:** 10.3389/fmicb.2017.02184

**Published:** 2017-11-07

**Authors:** Li Han, Yanfeng Peng, Yuangyuan Zhang, Wujiu Chen, Yuping Lin, Qinhong Wang

**Affiliations:** ^1^School of Food and Bioengineering, Zhengzhou University of Light Industry, Zhengzhou, China; ^2^CAS Key Laboratory of Systems Microbial Biotechnology, Tianjin Institute of Industrial Biotechnology, Chinese Academy of Sciences, Tianjin, China

**Keywords:** *Saccharomyces cerevisiae*, cytochrome P450, metabolic engineering, renewable sugar, fatty acids, α, ω-dicsarboxylic acids

## Abstract

Medium-chain (C8–C14) α, ω-dicarboxylic acids (α, ω-DCAs), which have numerous applications as raw materials for producing various commodities and polymers in chemical industry, are mainly produced from chemical or microbial conversion of petroleum-derived alkanes or plant-derived fatty acids at present. Recently, significant attention has been gained to microbial production of medium-chain α, ω-DCAs from simple renewable sugars. Here, we designed and created a synthetic omega oxidation pathway in *Saccharomyces cerevisiae* to produce C10 and C12 α, ω-DCAs from renewable sugars and fatty acids by introducing a heterogeneous cytochrome P450 CYP94C1 and cytochrome reductase ATR1. Furthermore, the deletion of fatty acyl-CoA synthetase genes *FAA1* and *FAA4* increased the production of medium-chain α, ω-DCAs from 4.690 ± 0.088 mg/L to 12.177 ± 0.420 mg/L and enabled the production of C14 and C16 α, ω-DCAs at low percentage. But blocking β-oxidation pathway by deleting fatty-acyl coenzyme A oxidase gene *POX1* and overexpressing different thioesterase genes had no significant impact on the production and the composition of α, ω-dicarboxylic acids. Overall, our study indicated the potential of microbial production of medium-chain α, ω-DCAs from renewable feedstocks using engineered yeast.

## Introduction

Medium-chain (C8-C14) α, ω-dicarboxylic acids (α, ω-DCAs) are used in the production of a variety of chemical products and intermediates, such as nylons and other polyamides, polyesters, resins, and perfumes (Song et al., 2013). Medium-chain α, ω-DCAs are mainly oxidized in mitochondria where they are transported through four different pathways and might be a suitable fuel substrate (Mingrone and Castagneto, 2006). Conventionally, medium-chain α, ω-DCAs are produced from petrochemical feedstocks or fatty acids by chemical processes which require high temperature and pressure, strong acids (H_2_SO_4_, HNO_3_), or toxic oxidants (ozone). The chemical synthesis methods of medium-chain α, ω-DCAs are harmful to our environment and cause serious ecological problems (Zhang et al., 2009; Köckritz and Martin, 2011). Thereby, it is very necessary to develop greener and sustainable processes to replace the present chemical processes, and more and more attention has been paid toward microbial production of medium-chain α, ω-DCAs via metabolic engineering and synthetic biology (Biermann et al., 2011; Seo et al., 2015).

Petrochemical feedstocks, such as dodecane and decane, could be oxidized to ω-fatty acid, then to α, ω-DCAs via widespread microbial degradation pathway of alkane (Craft et al., 2003; Wentzel et al., 2007). In China, microbial production of C11-C14 α, ω-DCAs from alkane has been industrialized. Similarly, fatty acids can also be ω-hydroxylated and then further oxidized to the corresponding diacids (Sanders et al., 2005, 2006), which are subjected to β-oxidation and eliminated in the urine in human and some animals (Kolvraa and Gregersen, 1986; Ferdinandusse et al., 2004). Fatty acids such as oleic acid, ricinoleic acid, and lesquerolic acid were recently found to be enzymatically cleaved into C9–C13 and C7–C9 carboxylic acids by naturally-occurring or recombinant microorganisms (Song et al., 2013, 2014). Recently, microbial production of medium-chain α, ω-DCAs from renewable glucose by engineering omega oxidation in *Escherichia coli* was reported (Bowen et al., 2016; Haushalter et al., 2017; Kallscheuer et al., 2017). By combining β-oxidation reversal and ω-oxidation pathway in *E. coli*, medium chain α, ω-DCAs (chain length from 6 to 10) could be produced with glycerol as feedstock (Clomburg et al., 2015). Furthermore, a wide range of small molecules, include α, ω-DCAs with chain length from 6 to 10, could be also produced from glucose in *E. coli* based on carbon and energy-efficient non-decarboxylative claisen condensation and subsequent β-reduction reactions (Cheong et al., 2016). Thus, greater commercial utility and sustainability might be achieved through microbial production of medium-chain α, ω-DCAs from abundant renewable lignocellulose as well as renewable plant oil resources.

The process from hydroxylation of terminal alkyl carbon (the carbon most distant from the carboxyl group of the fatty acid) of fatty acid to an alcohol, and then finally to a dicarboxylic acid is known as omega oxidation (ω-oxidation). Verkade and Van Der Lee (1934) first proposed ω-oxidation of fatty acids *in vivo* which almost occurs in fungi, higher plants and animals (Miura, 2013; Kim et al., 2016). The critical process of ω-oxidation is catalyzed by a hemoprotein cytochrome P450 enzyme which generally convert fatty acids to hydroxyfatty acids (Wanders et al., 2011). A large amount of cytochrome P450s with function of fatty acid hydroxylation has been reported from humans, plants and microbes, and most cytochrome P450s belong to membrane bound protein (Graham and Peterson, 1999; Williams et al., 2000). To produce medium-chain α, ω-DCAs by engineering ω-oxidation, cytochrome P450 which can catalyze medium-chain fatty acids would be necessary. Across nature, the substrate range of cytochrome P450s is vast and exceeds that of other enzymes. The range of different chemical transformations performed by P450s is also substantial, and continues to expand through interrogation of the properties of novel P450s, and selective hydroxylation can be undertaken on a highly functionalized molecule without the need for functional group protection (Lundemo and Woodley, 2015; Girvan and Munro, 2016). However, it is easy to find amounts of cytochrome P450s with function of fatty acid hydroxylation in nature, but high active cytochrome P450s for ω-oxidation of medium-chain fatty acids or hydrocarbons were not available (Scheps et al., 2011; Kim et al., 2016). Hence, both rational design and directed evolution approaches were developed to obtain efficient cytochrome P450s with higher substrate selectivity (Renault et al., 2007; Behrendorff et al., 2015).

**FIGURE 1:**
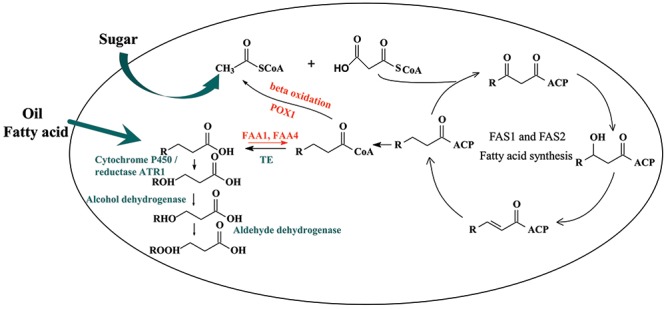
Biosynthesis of dicarboxylic acid by *Saccharomyces cerevisiae*. FAS1 and FAS2, fatty acid synthase; TE, acyl-CoA thioesterase; FAA1/FAA4, acyl-CoA synthetase.

In this work, we aimed to choose a suitable cytochrome P450 enzyme and then built a synthetic ω-oxidation pathway in *Saccharomyces cerevisiae* to produce medium-chain α, ω-DCAs. *S. cerevisiae* is a biological safe strain and has certain advantages to produce organic acid even at a low pH condition. Fermentation at a low pH condition could decrease the cost of acidification in the process of extracting (Abbott et al., 2009). Recently, many metabolic engineering strategies, including regulating the key genes of acetyl-CoA carboxylase (*ACC1*), fatty acid synthase 1 (*FAS1*) and fatty acid synthase 2 (*FAS2*) (Runguphan and Keasling, 2014), deleting the genes of fatty-acyl-CoA synthetase *FAA1*, *FAA4* (Chen et al., 2014; Runguphan and Keasling, 2014) and *FAT1*(Leber et al., 2015), and overexpressing some genes of acyl-CoA thioesterase (*TE*) (Chen et al., 2014; Leber and Da Silva, 2014; Li et al., 2014), have been developed to increase the production of fatty acids in *S. cerevisiae*. Other strategies such as blocking the β-oxidation (Leber et al., 2015; Lian and Zhao, 2015), reconstructing a neutral lipid recycle (Leber et al., 2015) and introducing a citrate lyase pathway (Zhou et al., 2016) were also used. Based on literature information, CYP94C1 has been reported as a wounding-responsive cytochrome P450 which could be able to catalyze fatty acids with varying aliphatic chain lengths (C12–C18) to hydroxyfatty acids and then to α, ω-DCAs (Kandel et al., 2007). Therefore, in this study, by only achieving functional expressions of heterogeneous P450 cytochrome *CYP94C1*/the corresponding cytochrome reductase gene *ATR1*, we combined the synthetic CYP94C1 dependent ω-oxidation with cellular fatty acid pathway to produce medium-chain α, ω-DCAs in *S. cerevisiae* with renewable sugar as substrate (**Figure 1**). Furthermore, the effect of the production of medium-chain α, ω-DCAs as well as their product spectrum was analyzed by improving the production of cellular fatty acid. Our study provides potential industrial strains for microbial production of medium-chain α, ω-DCAs from renewable feedstocks.

## Materials and Methods

### Strains, Plasmids, and Media

The yeast strains used in this study were constructed from *S. cerevisiae* BY4741 (**Table 1**). The yeast knockout strains of *S. cerevisiae* BY4741ΔPOX1 and BY4741ΔFAA1 were purchased from EUROSCAF. *S. cerevisiae* BY4741ΔFAA1ΔFAA4 knockout strain was constructed from *S. cerevisiae* BY4741ΔFAA1 by replacing *FAA4* with *HIS3* selection marker based on homologous recombination. The plasmids used in this study, which are listed in **Table 2**, were generated from pYES2/CT vector (Invitrogen). These plasmids contain the yeast 2 μ origin of replication, which results in yeast transformants with a high copy number of recombinant plasmids (10–40 copies per cell).

**Table 1:** *Saccharomyces cerevisiae* strains used in this study.

Strain name	Genotype	Reference
BY4741	Matα; his3Δ1; leu2Δ0; met15Δ0; ura3Δ0	EUROSCAF
BY4741ΔPOX1	Mat a; his3Δ1;leu2Δ0;met15Δ0;ura3Δ0; pox1::kanMX4	EUROSCAF
BY4741ΔFAA1	Mata; his3Δ1; leu2Δ0; met15Δ0; ura3Δ0; faa1::kanMX4	EUROSCAF
BY4741ΔFAA1ΔFAA4	Mata;his3Δ1;leu2Δ0; met15Δ0; ura3Δ0; faa1::kanMX4; faa4::HIS3	This study
Control	Matα;his3Δ1;leu2Δ0;met15Δ0;ura3Δ0;pYES2/CT	This study
CYP94	Matα;his3Δ1;leu2Δ0;met15Δ0;ura3Δ0; pYES-CYP94C1-ATR1	This study
CYP94ΔFAA1	Matα;his3Δ1;leu2Δ0; met15Δ0; ura3Δ0; faa1::kanMX4; pYES-CYP94C1-ATR1	This study
CYP94ΔFAA1ΔFAA4	Matα;his3Δ1;leu2Δ0; met15Δ0; ura3Δ0; faa1::kanMX4; faa4::HIS3; pYES-CYP94C1-ATR1	This study
CYP94ΔFAA1ΔFAA4 ACOT8	Matα;his3Δ1;leu2Δ0; met15Δ0; ura3Δ0; faa1::kanMX4; faa4::HIS3; pYES-CYP94C1-ATR1; pLEU2-GPD-ACOT8	This study
CYP94ΔFAA1ΔFAA4 PTE1	Matα;his3Δ1;leu2Δ0; met15Δ0; ura3Δ0; faa1::kanMX4; faa4::HIS3; pYES-CYP94C1-ATR1; pLEU2- GPD-PTE1	This study
CYP94ΔFAA1ΔFAA4 ‘TESA	Matα;his3Δ1;leu2Δ0; met15Δ0; ura3Δ0; faa1::kanMX4; faa4::HIS3; pYES-CYP94C1-ATR1; pLEU2- GPD-‘TesA	This study
CYP94ΔFAA1ΔFAA4 UCFATB	Matα;his3Δ1;leu2Δ0; met15Δ0; ura3Δ0; faa1::kanMX4; faa4::HIS3; pYES-CYP94C1-ATR1; pLEU2- GPD-UcfatB	This study

**Table 2:** Plasmids used in this study.

Name	Gene expressed	Plasmid type	Marker	Reference
pYES2/CT	None	2-micron replicon	*URA3*	Invitrogen
pRS313	None	centromere: CEN6	*HIS3*	ATCC
pYES2/CT’	None	2-micron replicon	*URA3*	This study
pYES-CYP94C1	*P_GAL1-_CYP94C1*	2-micron replicon	*URA3*	This study
pYES-ATR1	*P_GAL1-_ATR1*	2-micron replicon	*URA3*	This study
pYES-CYP94C1-ATR1	*P_GAL1-_CYP94C1, P_GAL1-_ATR1*	2-micron replicon	*URA3*	This study
pLeu2-GPD-mcherry	None	2-micron replicon	*LEU2*	Lab collection
pLeu2-GPD-ACOT8	*P_GPD-_ACOT8*	2-micron replicon	*LEU2*	This study
pLeu2-GPD-PTE1	*P_GPD-_PTE1*	2-micron replicon	*LEU2*	This study
pLeu2-GPD-‘TESA	*P_GPD-_TESA*	2-micron replicon	*LEU2*	This study
pLeu2-GPD-UcfatB	*P_GPD-_UcfatB*	2-micron replicon	*LEU2*	This study

Yeast and bacterial strains were stored in 30% glycerol at -80°C. *E. coli* was grown in Luria-Bertani medium at 37°C. Yeast strain without plasmid was cultivated in YPD medium (10 g/L yeast extract (OXOID), 20 g/L peptone (OXOID) and 20 g/L glucose). Yeast transformants were selected by either *HIS3*, *URA3* or *LEU2* auxotroph with yeast minimal medium [6.7 g/L of Yeast Nitrogen Base (Sigma), 20 g/L glucose, Yeast Synthetic Drop-out Medium Supplements without histidine, leucine, tryptophan and uracil (Sigma) and a mixture of appropriate amino acids]. Yeast cells were cultivated at 30°C.

*FAA4* knockout strain was generated from *S. cerevisiae* BY4741ΔFAA1 by homologous recombination using *HIS3* selectable marker replacement. Gene disruption cassettes containing the *HIS3* selectable marker was obtained by PCR of the pRS313 plasmid with primer 1 and 2. Oligonucleotide primers used for PCR, gene cloning, gene knockout in this study are included in Supplementary Table S1. Yeast cells were transformed using the LiAc/PEG method as previously described (Gietz and Schiestl, 2007). Following yeast transformations, colonies were selected on yeast minimal medium lacking uracil and/or histidine and confirmed via PCR and sequencing. The PCR identification of the deletion of FAA4 used primers 3 and 4.

### Plasmid Construction

To obtain plasmids pYES-CYP94C1, pYES-ATR1 and pYES-CYP94C1-ATR1 (**Table 2**), *CYP94C1* and *ATR1* were amplified from *Arabidopsis thaliana* cDNA using primer pair 5, 6 and primer pair 7, 8. The Kozak sequence AAACA was added 5′ of the start codon to enhance expression (Hamann and Møller, 2007). To construct cassette CYP94C1 and cassette ATR1 in the same plasmid, *Bgl*II and *Sal*I restriction sites were, respectively, introduced into pYES2/CT at the 5′ end of the *GAL1* promoter and 3′ end of the *CYC1* terminator. To achieve this, the DNA sequence of the plasmid pYES2/CT without the sequence between promoter *P*_GAL1_ and terminator *T*_CY C1_ was amplified with primer 9 and 10 (containing *Bgl*II and *Sal*I), and then the expression cassette between PGAL1 and TCYC1 was amplified with primer 11 and 12 (containing *Bgl*II and *Sal*I). Both amplified products were digested with *Bgl*II and *Sal*I, and then were ligated to construct pYES2/CT’. The amplified product CYP94C1 was digested with *Sac*I and *Xho*I, and cloned into pYES2/CT’ to construct pYES-CYP94C1. For integration of an expression cassette for *ATR1*, amplified *ATR1* was digested with *Kpn*I and *EcoR*I, and cloned into pYES2/CT to construct pYES-ATR1. The expression cassette P_GAL1_-ATR1-T_CY C1_ was amplified with primers 13 and 14 which contain *Sal*I restriction site and then digested by *Sal*I and cloned into pYES-CYP94C1 to obtain plasmid pYES-CYP94C1-ATR1.

To co-express thioesterase, plasmid pLeu2-GPD-mcherry previously constructed from pRS425 by our lab was used, and this plasmid contained 2 μ origin of replication and *mCherry* between promoter *P_GPD1_* and terminator *T*_CY C1_. The plasmid pLeu2-GPD-mcherry also contains *leu2d* allele of a leucine biosynthetic gene *LEU2* for auxotrophic selection by the media without leucine. To construct the recombinant plasmids containing thioesterase, pYES-LEU2-ACOT8, pYES-LEU2-PTE1, pYES-LEU2-‘TESA and pYES-LEU2- UcfatB, all the thioesterase genes digested by *Spe*I and *Hind*III and then were cloned into pLeu2-GPD-mcherry to replace *mCherry* with corresponding thioesterase gene. *Acot8* gene was synthesized based on the CDS of accession No.NM_133240 and then cloned by primer 15 and 16. *PTE1* was cloned with primer 17 and 18 from *S. cerevisiae* BY4741. *UcfatB* gene was synthesized based on the CDS of accession No.M94159. *‘TesA* was cloned by primer 19 and 20 from *E. coli* MG1655.

### *In Vivo* Analysis of Conversion of Medium-Chain Fatty Acids by *S. cerevisiae*

*In vivo* analysis of conversion of medium-chain fatty acids by engineered *S. cerevisiae* was referred to the method from literature (Pompon et al., 1996) with some modifications. A fresh single colony was inoculated in 5 mL yeast minimal medium, and the overnight culture was then inoculated into 50 mL YPEG medium (1% yeast extract, 2% Peptone, 2% glycerol, 1% ethanol, 2% glucose) in 250 mL flask to achieve an initial OD600 of 0.05. When the OD600 achieved to 1.3–1.5 after incubation, 2% galactose was added to the medium to induce the expression of CYP94C1 and *ATR1*. After further incubation of 12 h, 10 mL of induced yeast cultures were collected by centrifugation with 4000 *g* for 5 min and the pellets were resuspended in 10 mL YPL medium (1% Peptone, 0.5% Yeast Extract, 0.5% NaCl, 0.4% Na_2_HPO_4_, pH 7.0) in 50 mL flask and 1 mM substrate of hexanoic acid, octanoic acid, decanoic acid, or dodecanoic acid was added to the culture to start the transformation, respectively. After 3 h, 6 h and 22 h incubation, 1 mL sample was added 160 μL 6M HCl and extracted by 2 mL ethyl acetate for twice. The extract liquid was dried by Pressure Blowing Concentrator and dissolved in 150 μL ethyl acetate. Then, 150 μL derivative reagents [99:1 = Bis(trimethylsilyl)trifluoroacetamide (BSTFA): Trimethylsilyl (TMS)] was used to for derivatization on 90°C about 50 min (Nakano et al., 2009). Derivative products were analyzed by GC-MS (Agilent 5975MS/7890A, chromatographic column Agilent HP-5MS). The GC program of medium-chain α, ω-DCAs and hydroxyfatty acid was as follows: an initial temperature of 80°C was maintained for 2 min, followed to 130°C at a rate of 40°C/min, held for 1 min; 4°C/min rate to 210°C, held for 1min; 30°C/min to 290°C, held for 1 min. Split ratio was 10:1, injection volume 1 μL (Nakano et al., 2009).

### *In Vitro* Analysis of Conversion of Medium-Chain Fatty Acids by *S. cerevisiae*

*In vitro* analysis of conversion of medium-chain fatty acids by *S. cerevisiae* was also referred to the method from literature (Pompon et al., 1996) with some modifications. A fresh single colony was inoculated into 50 mL yeast minimal medium for about 24 h. Then a 1:50 dilution was made into YPGE medium and grown on 30°C until cell density reached 8 × 10^7^ cells per mL (about 24–36 h). After that, induction was initiated by addition of 2% galactose for 8–15 h until the cell density reached to 2–5 × 10^8^ cells per mL. Cells were harvested by centrifugation at 7000 *g* for 5 min at 4°C. The collected cells were washed with ice-cold 50 mM Tris-HCl buffer (pH 7.4) for twice and then re-suspended in 50 mM ice-cold Tris-HCl buffer containing 40% glycerol, 5 mM DTT and 1 mM EDTA (pH 7.4). The cells were mechanically disrupted with glass beads (0.5–0.8 mm) in the cold room. The disrupted cells were diluted with 50 mM Tris-HCl containing 1 mM EDTA and 0.6 M sorbitol (pH 7.4) and centrifuged at 5000 *g* for 5 min at 4°C. The supernatant was centrifuged again at 10000 *g* for 20 min at 4°C to remove the soluble protein fraction and repeated again. Then, the supernatant was centrifuged at 20000 *g* for 20 min after adding 20 mM CaCl_2_ to obtain the microsome in the precipitate. The microsome was then dissolved in ice-cold 50 mM Tris-HCl buffer containing 0.4 M sorbitol. Analysis of *in vitro* enzyme activity was operated in 0.2 M potassium phosphate buffer (pH 7.4). The reaction system contained 0.5 mM NADPH, 0.5 mM DTT, NADPH regeneration system with 3 mM 6-glucose-6-phosphate and 0.5 U 6-glucose-6-phosphate dehydrogenase, 0.5 mg microsome, and 50 μM of substrate hexanoic acid, octanoic acid, or decanoic acid. The reaction was proceeded on 30°C and 200 μL of sample was taken for every 1 h and analyzed by GC-MS. The samples were added 6 M HCl and then extracted by 1 mL ethyl acetate twice. The extract liquid were dried by Pressure Blowing Concentrator and dissolved with 150 μL ethyl acetate, and then 150 μL derivative reagents (99:1 = BSTFA:TMS) was used to for derivatization on 90°C about 50 min. The GC program was the same as *in vivo* analysis method above.

### GC-MS Analysis of Medium-Chain α, ω-DCAs, Hydroxyfatty Acid, and Free Fatty Acids in Fermentation Broth

For production of medium-chain α, ω-DCAs, a single colony was inoculated in 5 mL yeast minimal medium and the overnight culture was used to inoculate 50 mL fermentation medium [6.7 g/L of Yeast Nitrogen Base, 0.2% glucose, 2% galactose, and Yeast Synthetic Drop-out Medium Supplements without histidine, leucine, tryptophan and uracil (Sigma) with appropriate mixture of amino acids] in 150 mL flask to achieve an initial OD_600_ of 0.05. After 24, 36, and 108 h-incubation, 1 mL of yeast culture was taken for GC-MS analysis.

To quantitatively analyze the content of medium-chain α, ω-DCAs and ω-hydroxyfatty acids, internal standard methods was chosen. 15-hydroxypentadecanoic acid was as the internal standard and parameter SIM m/z of 55 was chosen to be characteristic peak for quantitative analysis. Standards containing hexadecanedioic acid, 16-hydroxyhexadecanoic acid, tetradecanedioic acid, 14-hydroxytetradecanoic acid, dodecanedioic acid, 12-hydroxydodecanoic acid, decanedioic acid and 10-hydroxydecanoic acid were dissolved in ethyl acetate to be mixture of 1 g/L. Then the 1 g/L mixture was diluted to be 250, 125, 62.5, 31.25, 6.25, and 3.125 mg/L standard solution. 120 μL standard solution and 30 μL internal standard of 50 mg/L15-hydroxypentadecanoic acid dissolved in ethyl acetate were mixed, and then 150 μL derivatization reagent BSTFA:TMS (99:1) was added to proceed derivatization treatment on 90°C for 50 min. The standard solution was cooled down to room temperature and filtered through 0.22 μm membrane for GC-MS analysis. 1 mL sample of yeast culture was treated as above for GC-MS analysis. The GC program was similar with *in vivo* analysis method above but with some modifications convenient for long chain dicarboxylic acids and hydroxyfatty acids analysis (Nakano et al., 2009): an initial temperature of 100°C was maintained for 2 min, followed to 200°C at a rate of 40°C/min, held for 1 min; 4°C/min rate to 245°C, held for 1 min; 30°C/min to 290°C, held for 1 min. Split ratio was 10:1, injection volume 1 μL.

### GC-MS Analysis of Decanoic Acid and Dodecanoic Acid Conversion by Engineered Strains

To analyses the conversion rate of decanoic acid and dodecanoic acid by different engineered yeast, the conversion products of decanedioic acid or dodecanedioic acid and the unconverted decanoic acid or dodecanoic acid were quantified by GC-MS to calculate the conversion rates. Conversion rate equal to per mole of fatty acids converted into molar number of dicarboxylic acids. Octanoic acid was chosen as the internal standard of fatty acids, and 15-hydroxypentadecanoic acid was chosen as the internal standard of medium-chain α, ω-DCAs and hydroxyfatty acids. The transformation process was same as *in vivo* analysis of enzyme activity of engineered strains above.

## Results

### Expression and Activity of Cytochrome P450 *CYP94C1* with *ATR1* toward Medium-Chain Fatty Acids in *S. cerevisiae*

**FIGURE 2:**
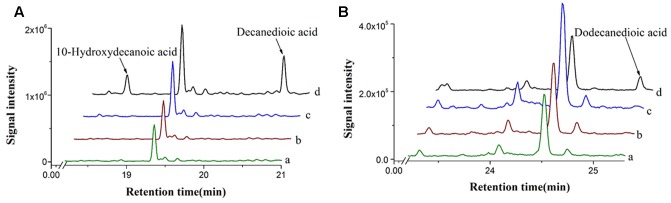
*In vivo* enzyme characterization with different fatty acids in *S. cerevisiae* expressing *CYP94C1* and *ATR1*. **(A)** Decanoic acid and **(B)** Dodecanoic acid. **(a)** BY4741 with control plasmid pYES2/CT. **(b)** BY4741 with plasmid pYES-ATR1. **(c)** BY4741 with plasmid pYES-CYP94C1. **(d)** BY4741 with plasmid pYES-CYP94C1-ATR1 (CYP94).

A cytochrome P450 enzyme CYP94C1 from *A. thaliana*, which displays a broad substrate specificity on fatty acids (C12–C18), has been reported (Kandel et al., 2007). CYP94C1 along with a cytochrome reductase could convert fatty acids to hydroxyfatty acids and then to dicarboxylic acids. To identify whether linear and medium-chain length of fatty acids could be catalyzed by CYP94C1, we then co-expressed both *CYP94C1* and the cytochrome reductase gene *ATR1* under promoter *P*_GAL1_ in *S. cerevisiae* and prepared the microsomes from the engineered yeast for activity characterization. Medium-chain linear fatty acids with even carbon chain from C6 to C12 were chosen to be detected. CYP94C1 only showed the activity to C10 and C12 fatty acids, but not to C6 and C8 fatty acids. Based on the results shown in **Figure 2**, decanoic acid (C10 fatty acid) could be converted to 10-hydroxydecanoic acid and then to decanedioic acid while dodecanoic acid (C12 fatty acid) was directly converted to dodecanedioic acid. Our study confirmed that shorter linear C10 fatty acid (decanoic acid) should be the substrate of CYP94C1, which was not reported in the literature (Kandel et al., 2007). In our study, only dodecanedioic acid, but no 12-hydroxydodecanoic acid was detected. This result implicated that the reaction of 12-hydroxydodecanoic acid to dodecanedioic acid could be too fast to detect the intermediate product. Furthermore, we analyzed the activity of CYP94C1 on decanoic acid via *in vitro* experiments, and 10-hydroxydecanoic acid was also identified (Supplementary Figure S1).

### Production of Medium-Chain α, ω-DCAs from Renewable Sugars by Engineered *S. cerevisiae*

**FIGURE 3:**
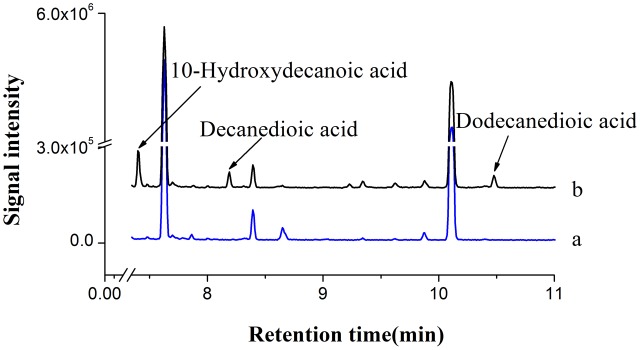
GC-MS analysis of fermentation products. **(a)** BY4741 with control plasmid pYES2/CT. **(b)** BY4741 with plasmid pYES-CYP94C1-ATR1 (CYP94). The ion spectra of these compounds were shown in Supplementary Figure S2.

Based on our study and literature information (Kandel et al., 2007), C10 to C18 fatty acids could be converted to the corresponding α, ω-dicarboxylic acids by co-expressing *CYP94C1* and *ATR1* in *S.cerevisiae*. Thus, we considered the possibility of combining CYP94C1-dependent omega oxidation pathway with endogenous fatty acid synthetic pathway in engineered *S. cerevisiae* for producing medium-chain α, ω-DCAs from renewable sugars. Our results showed that CYP94C1 combined with ATR1 in *S. cerevisiae* could be used to convert fatty acids to relatively stable end products of α, ω-dicarboxylic acids. Hence, we used the engineered *S. cerevisiae* strain CYP94 (**Table 1**) to verify whether medium-chain α, ω-DCAs could be produced in fermentation medium containing 2% galactose and 0.2% glucose. After 24 h-fermentation, 10-hydroxydecanoic acid was detected in fermentation broth, and then decanedioic acid and dodecanedioic acid were also detected in 36 h, meanwhile these products were not found in fermentation broth of control strain (**Figure 3**). Therefore, we can combine cytochrome P450 mediated omega oxidation pathway with yeast endogenous fatty acid synthetic pathway to succesfully produce medium-chain α, ω-DCAs in engineered *S.cerevisiae*. The long-chain saturated fatty acids, hexadecanoic acid (C16) and octadecanoic acid (C18) were main products in *S. cerevisiae* (Tehlivets et al., 2007) and CYP94C1 could catalyze fatty acid with chain length from C12 to C18, but long-chain α, ω-DCAs such as hexadecanedioic acid and octadecanedioic acid were not detected in strain CYP94 after 36 h in our study. We speculated that CYP94C1 favored to catalyze medium-chain fatty acid, and this is consistent to the literature report that CYP94C1 showed higher specicity of fatty acid C12 than that of other longer chain fatty acids C14–C18 (Kandel et al., 2007).

### Effect of *POX1* Deletion in β-Oxidation Pathway on Accumulation of Medium-Chain α, ω-DCAs

In organisms, fatty acids will be broken down to acetyl-CoA by β-oxidation pathway. While in some animals, long chain dicarboxylic acids could be degraded into short chain dicarboxylic acids, such as adipic acid or succnic acid, by β-oxidation. To decrease the consumption of the precursors of fatty acids and thereby increase the end products of dicarboxylic acids, we were going to block β-oxidation pathway for improving the production of medium-chain α, ω-DCAs. *POX1*, the key gene in the β-oxidation pathway, encodes acyl-CoA oxidase which oxidizes acyl-CoA to *trans*-2-enoyl-CoA after fatty acids are transported to peroxisome. To block β-oxidation pathway, *POX1* was deleted first and the resulting deletion strain (CYP94ΔPOX1) produced 4.234 mg/L hydroxyfatty acids and dicarboxylic acids, which was not significantly different from the production of 4.690 mg/L by the control strain (CYP94) (**Table 3**). Thus, the deletion of *POX1* did not improve the production of dicarboxylic acids. As it was reported that the deletion of *POX1* did not improve the accumulation of fatty acids and fatty acids derived lipids (Runguphan and Keasling, 2014), our result also showed the deletion of *POX1* was not effective to improve the accumulation of the dicarboxylic acids.

**Table 3:** Production of ω-hydroxyfatty acid and α, ω-dicarboxylic acid with engineered *S. cerevisiae* strains.

Strain	Plasmid	Total hydroxyfatty acid and dicarboxylic acid (mg/L)
CYP94	pYES-CYP94C1-ATR1	4.690 ± 0.088
CYP94 ΔPOX1	pYES-CYP94C1-ATR1	4.234 ± 0.092
CYP94 ΔFaa1	pYES-CYP94C1-ATR1	7.717 ± 0.009
CYP94 ΔFaa1ΔFaa4	pYES-CYP94C1-ATR1	12.177 ± 0.420
CYP94ΔFaa1ΔFaa4 Acot8	pYES-CYP94C1-ATR1, pLEU2-GPD-acot8	11.220 ± 0.126
CYP94ΔFaa1ΔFaa4 PTE1	pYES-CYP94C1-ATR1, pLEU2-GPD-PTE1	11.295 ± 0.367
CYP94ΔFaa1ΔFaa4 ‘TesA	pYES-CYP94C1-ATR1, pLEU2-GPD-‘TesA	11.130 ± 0.783
CYP94ΔFaa1ΔFaa4 UcfatB	pYES-CYP94C1-ATR1, pLEU2-GPD-UcfatB	12.133 ± 0.184

### Effect of Acyl-CoA Synthetase Gene Deletion on Production of Medium-Chain α, ω-DCAs

In *S. cerevisiae*, free fatty acids first need to be activated to CoA form by acyl-CoA synthetase and then to be degraded to acetyl-CoA through β-oxidation pathway. There are five acyl-CoA synthetase genes in *S. cerevisiae*, including *FAA1-4* and *FAT1*, and FAA1 and FAA4 are reponsible for the main activity of acyl-CoA synthetase (Trotter, 2001). Although *POX1 deletion* did not improve the production of dicarboxylic acids in our study, deletion of *FAA1* and *FAA4* has been reported to increase the production of free fatty acids in *S. cerevisiae* (Runguphan and Keasling, 2014). Hence, we deleted *FAA1* and *FAA4* to analyze their effects on the production of dicarboxylic acids and hydroxyfatty acids.

**FIGURE 4:**
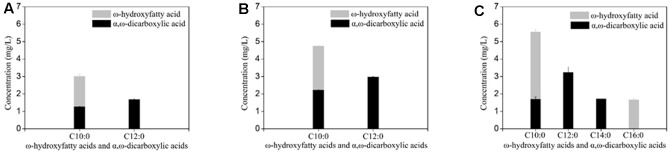
ω-hydroxyfatty acid and α, ω-dicarboxylic acid analysis with engineered *S. cerevisiae* after fermentation about 36 h. **(A)** CYP94. **(B)** CYP94ΔFaa1. **(C)** CYP94 ΔFaa1ΔFaa4. The error bars represent the average from three independent experiments.

Deletion of *FAA1* in engineered BY4741 co-expressing *CYP94C1* and *ATR1* resulted in an increase of the production to 7.717 mg/L of hydroxy fatty acids and dicarboxylic acid after fermentation about 36 h (**Table 3**). Compared with the control strain (CYP94), the *FAA1* deletion strain showed the significant increase of the production of hydroxyfatty acids and dicarboxylic acids (**Figures 4A,B**). Deletion of both *FAA1* and *FAA4* in *S.cerevisiae* further increased the total production of hydroxyfatty acids and dicarboxylic acids to 12.177 mg/L (**Table 3**), and longer-chain dicarboxylic acids and hydroxyfatty acids such as tetradecanedioic acid and 16-hydroxyhexadecanoic acid were accumulated after 36 h (**Figure 4C**). Therefore, we speculated that deletion of *FAA1* and *FAA4* increased the accumulation and secretion of free fatty acids, which in succession improved the production of hydroxyfatty acids and dicarboxylic acids. Since CYP94C1 is able to catalyze linear chain fatty acids from C10–C18, longer-chain hydroxyfatty acids and dicarboxylic acids were thus produced with the increased production of fatty acids.

**FIGURE 5:**
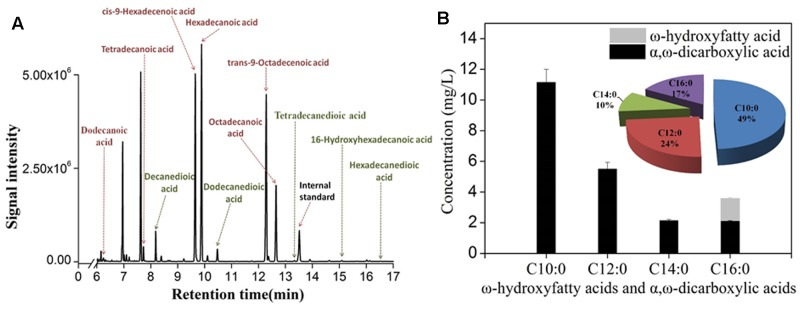
GC-MS profile of fermentation products from engineered strain CYP94ΔFaa1ΔFaa4 after 108 h-fermentation **(A)** and the production and percentage of ω-hydroxyfatty acids and dicarboxylic acids **(B)**. The error bars represent the average from three independent experiments.

As deletion of *FAA1* and *FAA4* improved the overall production of hydroxyfatty acids and dicarboxylic acids, we further analyzed the fermentation products by GC-MS. After 36 h fermentation, the engineered strain with *FAA1* and *FAA4* deletion was able to convert majority of free fatty acids to hydroxyfatty acids and then to dicarboxylic acids, but still accumulated small amount of fatty acids, inclduing dodecanoic acid (C12 fatty acid), tetradecanoic acid (C14 fatty acid), *cis*-9-hexadecenoic acid (16:1(9c) fatty acid), hexadecanoic acid (C16 fatty acid), *trans*-9-Octadecanoic acid (C18:1(9t) fatty acid) and octadecanoic acid (C18 fatty acid) (data was not shown). At the long ferementaiton time (108 h), some amount of residual linear fatty acids such as C14 and C16 fatty acids were still detected (**Figure 5A**). The total production of hydroxyfatty acids and dicarboxylic acids was about 22 mg/L including decanedioic acid (11.6 mg/L), dodecanedioic acid (5.8 mg/L), tetradecanedioic acid (2.0 mg/L), 16-hydroxyhexadecanoic acid and hexadecanedioic acid (3.6 mg/L) (**Figure 5B**). From the result at 108 h-fermentation, most hydroxyfatty acids were converted to dicarboxylic acids with only a few 16-hydroxyhexadecanoic acid (**Figure 5B**). Although some amount of linear fatty acids such as tetradecanoic acid (C14) and hexadecanoic acid C16) were accumulated, long-chain dicarboxylic acids were not increased and more medium-chain α, ω-DCAs such as decanedioic acid and dodecanedioic acid were produced. The ratio of decanedioic acid and dodecanedioic acid was 74% in the total amount of the hydroxyfatty acids and dicarboxylic acids (**Figure 5B**).

### Effect of Different Thioesterase Overexpression on Production of Medium-Chain α, ω-DCAs

Thioesterase exhibits the activity of splitting of an ester into acid and alcohol. Hence, overexpression of thioesterase often facilitate the production of fatty acid in yeast (Chen et al., 2014; Leber and Da Silva, 2014; Leber et al., 2016). For example, *‘TesA* in *E. coli* exhibits both acyl-ACP and acyl-CoA thioesterase and *‘TesA* could be actively expressed in *S.cerevisiae* to produce free fatty acids (Runguphan and Keasling, 2014). Since the fatty acid could be converted into dicarboxylic acid in presence of ω-oxidation (**Figure 1**), the production of dicarboxylic acid might increase if the production of fatty acid increased. To test this speculation, we chose different thioesterases, including two thioesterases from eukaryotic organism which are able to convert short fatty acyl-CoA, such as *Acot8* from mouse (Westin, 2005) and *UcfatB* from *Umbellularia californica* (Torella et al., 2013), and one thioesterase *PTE1* from *S. cerevisiae* (Maeda et al., 2006) as well as *‘TesA* from *E. coli* to investigate the effect on the production of dicarboxylic acids in engineered *S. cerevisiae* (strain CYP94ΔFaa1ΔFaa4). For the tesed thioesterase in our study, Acot8 (Westin, 2005) acts on fatty acyl-CoA with chain length less than 6, and Ucfat8 (Torella et al., 2013) with chain length less than 10.

After 36 h-feremenation, the engineered strains with overexpression of thioesterase did not improve the overall production of dicarboxylic acids further (**Table 3**). Subsequently, we explored the influence of different thioesterase on the percentage of each dicarboxylic acid. After 108 h-fermentation, overexpression of thioesterase also did not change the percentage of medium-chian dicarboxylic acid obviously compared with the control strain (CYP94ΔFaa1ΔFaa4) (**Figure 5** and Supplementary Figure S3). While the percentage of long-chain dicarboxylic acids such as hexadecanedioic acid varied from 12 to 22%, with PTE1 the most and Acot8 the least (Supplementary Figure S3).

### Conversion of Fatty Acids to Dicarboxylic Acids by Engineered *S. cerevisiae*

The engineered *S. cerevisiae* with CYP94C1 and ATR1 in the study could produce dicarboxylic acids from renewable sugars (**Table 3**) while the microsomes from engineered *S. cerevisiae* expressing CYP94C1 and its reductase was able to convert decanoic acid and dodecanoic acid to decanedioic acid and dodecanedioic acid, respectively, from our results (**Figure 2**). Hence, we compared the conversion effect of decanoic acid and dodecanoic acid to dicarboxylic acids by whole cell transformation with our engineered *S. cerevisiae*. Considering the toxicity of decanoic acid and dodecanoic acid to the cell of *S. cerevisiae*, low concentration (1 mM) of substrates were added to the convesion solution. Interestingly, when galactose was added to the conversion solution for inducing the overexpression of cytochrome P450 CYP94C1 and starting the whole cell transformation, most decanoic acid and dodecanoic acid were converted to hydroxyfatty acids by engineered *S. cerevisiae* with CYP94C1 and ATR1 (Strain CYP94) (Supplementary Figure S4). However, almost all fatty acids were converted to the end product dicarboxylic acids if galactose was not added (Supplementary Figure S4). The reason about this should be further investigated.

Nevertheless, to poduce dicarboxylic acids from fatty acids, the whole cell transformation without galactose supplement was selected. After 12 h incubation, substrate consumption of decanoic acid and dodecanoic acid were 74.357 and 75.360%, respectively, and the conversion rate of substrates to decanedioic acid and dodecanedioic acid was 2.638 and 0.693%, respectively, in engineered *S.cerevisiae* with CYP94C1 and ATR1 (**Table 4**). We also tested the capability of the engineered *S. cerevisiae* with both *FAA1* and *FAA4* deletion (strain CYP94ΔFaa1ΔFaa4) for converting fatty acid to dicarboxylic acids. The consumption rate of decanoic acid and dodecanoic acid was 74.312 and 83.679%, respectively, in CYP94ΔFaa1ΔFaa4. The consumption rate of fatty acid was very similar to that of engineered strain CYP94 without deletion, but the conversion rate of substrates to decanoic acid and dodecanoic acid was 9.107 and 2.890%, respectively. The conversion rate of fatty acids to dicarboxylic acids increased more than 3.4 times (**Table 4**). Though the deletion of *FAA1* and *FAA4* improved the conversion rate of fatty acids, most fatty acids were still consumed by the cell, and not converted to the dicarboxylic acids. How to improve the conversion rate of fatty acids to dicarboxylic acids by engineered *S. cerevisiae* should be further figured out for improving the production efficiencey.

**Table 4:** Conversion rate of decanoic acid and dodecanoic acid after 12 h with engineered *S. cerevisiae* strains.

Strain	Decanoic acid	Decanoic acid	Dodecanoic acid	Dodecanoic acid
	(consumption) %	(conversion rate) %	(consumption) %	(conversion rate) %
CYP94	74.357 ± 4.070	2.638 ± 0.171	75.360 ± 2.632	0.693 ± 0.006
CYP94ΔFaa1ΔFaa4	74.312 ± 6.077	9.107 ± 0.977	83.679 ± 1.875	2.890 ± 0.176

## Discussion

### Improving Expression of Cytochrome P450 in *S. cerevisiae*

In this study, we constructed plasmid pYES-CYP94C1-ATR1 with cytochrome P450 gene *CYP94C1* and cytochrome reductase gene *ATR1*, respectively, with promoter *P*_GAL1_ and terminator *T*_CY C1_ (Supplementary Figure S5). The engineered *S. cerevisiae* with *CYP94C1* and *ATR1* was able to produce hydroxyfatty acids and dicarboxylic acids. While the arrangement of gene cassette could increase the unstability of the recombinant plasmid since the recombination may occour due to the repeated sequence of promoter *P*_GAL1_ and terminator *T*_CY C1_. Though the similar arrangement of genes in plasmids from Westfall’s report (Westfall et al., 2012) did not influence the production of artemisinic acid, the reversal of *P_GAL1_-ADS-T_PGK1_* expression cassette was able to avoid the unstability of the plasmids and the genes on plasmids would not change easily as cell division. Some reports mentioned that integrating cytochrome reductase into the genome of *S. cerevisiae* faciliated to express variable cytochrome P450s and decreased the burden of cell to express large plasmids (Humphreys et al., 1999; Sauveplane et al., 2009). Hence, we should consider to reconstruct the expression cassette with different arrangement or integrate cytochrome reductase into the yeast genome for improving the stability of engineered strains.

As the promoter *P*_GAL1_ need galactose for induction and is depressed by glucose, this not only affected the production of targets, but also increased production cost. Since *GAL1*, *GAL10*, and *GAL7* in *S. cerevisiae* could be deleted to eliminate the depression of glucose to the *P*_GAL1_, thus glucose could be the carbon source and few galactose shoud be supplemented in the process of fermentation (Westfall et al., 2012). Moreover, Westfall’s report also mentioned that the deletion of *GAL80* resulted in no need of galactose to induce the promoter of *P*_GAL1_ (Westfall et al., 2012). Therefore, to reduce the fermentation cost, *P*_GAL1_ could be used based on some genetic modification to eliminate the depression of glucose. Of course, some other strong constitutive promoters could also be used to replace the promoter of *P*_GAL1_.

### Improving Production of Medium-Chain α, ω-DCAs and Product Spectrum

The production of hydroxyfatty acids and dicarboxylic acids in our study is still very low. How to improve the production of the products is a big challenge. Probably, we should not only improve the precursors of free fatty acids, but also enhance the efficiency of ω-oxidation pathway to redirect the metabolic flux to the synthesis of dicarboxylic acids. To improve the production of the precursors of free fatty acids, in the study, almost no effect was shown on the production of the dicarboxylic acids by deleting *POX1* to block the β-oxidation pathway of fatty acids. When deleting *FAA1* and *FAA4*, the production of dicarboxylic acids was indeed improved, but not significantly. Through the analysis of bioconversion of decanoic acid and dodecanoic acid to dicarboxylic acids with our engineered *S. cerevisiae*, we found that large amount of fatty acids were consumed by cell and only small part has been converted to dicarboxylic acids even after *FAA1* and *FAA4* deletion. Therefore, the consumption of fatty acids by the yeast cell resulted in low production of dicarboxylic acids. How to decrease the consumption of fatty acids by the cell and improve the production of free fatty acids is still main task for improving the production of dicarboxylic acids. To improve the production of free fatty acids, some strategies were developed. Zhou et al. (2016) has reported interrupting free fatty acid activation, introducing a synthetic chimeric citrate lyase pathway and expressing a FAS from *Rhodosporidium toruloides* in *S. cerevisiae* would significantly increase the production of free fatty acids. In addition, since hydroxyfatty acids were still detected after 108 h fermentation in our study, alcohol dehydrogenase and aldehyde dehydrogenase should probably be overexpressed in the engineered *S. cerevisiae* for enhancing the efficiency of omega oxidation.

In our work, most dicarboxylic acids are medium chain which contain carbon chain length of 10 and 12, such as decanedioic acid and dodecanedioic acid, but long-chain dicarboxylic acid such as hexadecanedioic acid was produced after *FAA1* and *FAA4* deletion. In order to control the chain length of dicarboxylic acids and increase the production of medium-chain α, ω-DCAs, we tested the effect of thioesterase which can transform short fatty acyl-CoA to short-chain fatty acids. While the additional expression of short-chain thioesterase such as *Acot8* and *UcfatB* did not improve the production of medium-chain α, ω-DCAs significantly and also not change the product spectrum of variable dicarboxylic acids in our study. Leber and Silva’s (2014) report mentioned that replacement of *FAS2* with *hFAS2* from human and expression of thioesterase *CpFatB* catalyzing short fatty acyl-CoA to short fatty acids in *S. cerevisiae* produced only about 68 mg/L of short fatty acids. The expression of thioesterase cannot produce large amount of short chain fatty acids in *S. cerevisiae* due to the special structure of *FAS* in *S. cerevisiae*. To solve this problem, Gajewski et al. (2017) rationally redesigned the FAS by minimally invasive protein engineering to obtain short chain fatty acids, such as hexanoic acid (C6) and octanoic acid (C8) as the main product (Gajewski et al., 2017). Hence, to obtain medium chain fatty acids, similar redesign of FAS by protein engineering should be tested.

To improve the product spectrum, choosing suitable cytochrome P450s which favor to medium-chain fatty acids or display better substrate specificity to decanoic acid or dodecanoic acid should be another choice. Certainly, rational design or directed evolution of CYP94C1 for improving substrate specificity was also the possible strategy. Arnold group at Caltech showed many success examples for improving substrate specificity by directed evolution of P450 BM3 over the past 10 years (Glieder et al., 2002; Arnold, 2015). Therefore, improving the production of fatty acids as well as controlling the chain length of target products should be considered to improve the production of medium chain dicarboxylic acids (Zhou et al., 2016).

The engineered *S. cerevisiae* overexpressing *CYP94C1* and *ATR1* could catalyze fatty acids such as decanoic acid and dodecanoic acid to produce dicarboxylic acids, but the conversion rate was very low as most fatty acid substrates was consumed by the cell. Therefore, how to decrease the fatty acids consumption should be solved. In this study, after blocking β-oxidation via *POX1* deletion, fatty acids were consumed but no dicarboxylic acids were produced. β-oxidation pathway happens in peroxisome and *S. cerevisiae* is able to grow in the medium with fatty acids as single carbon sources (Hiltunen et al., 2003; van Roermund et al., 2003). Though β-oxidation pathway was blocked, we speculated that the accumulation of fatty acyl-CoA in peroxisome was toxic to the cell of yeast and thus influence the normal ω-oxidation for conversing fatty acids to dicarboxylic acids as acyl-CoA synthetase could convert fatty acids to fatty acyl-CoA and then transported to peroxisome by PXA1/PXA2. To solve this problem, deleting both acyl-CoA synthetase gene *FAA1* and *FAA4* and acyl-CoA oxidase gene *POX1* should be tested. In addition, deletion of acyl-CoA synthetase gene *FAT1* and *FAA2* would block the transformation of fatty acids to fatty acyl-CoA and then decreased the fatty acids consumption (Leber et al., 2015). Hence, *FAT1* and *FAA2* might be suitable targets for decreasing the fatty acids consumption. What’s more, other fatty acyl-CoA synthetases in *S. cerevisiae* such as *FAA3* and *FAT2* (Black and DiRusso, 2007) could also be considered. As oils and fat could be converted to free fatty acids by lipase, so medium-chain α, ω-DCAs could be produced from oils by engineered *S. cerevisiae* if the conversion rate of fatty acids to dicarboxylic acids was improved significantly.

## Author Contributions

QW and LH designed the experiments. LH, YP, YZ, and WC performed the experiments. LH, YL, and QW analyzed the data and wrote the manuscript. All authors reviewed the manuscript.

## Conflict of Interest Statement

The authors declare that the research was conducted in the absence of any commercial or financial relationships that could be construed as a potential conflict of interest.
