# Supplementary material for: Designing and Creating a Synthetic Omega Oxidation Pathway in Saccharomyces cerevisiae Enables Production of Medium-Chain α, ω-Dicarboxylic Acids
Source: Front Microbiol. 2017 Nov 7;8:2184. doi: 10.3389/fmicb.2017.02184 (PMC5673993; doi:10.3389/fmicb.2017.02184)
Supplement: Supplementary file 1 [file Table_1.PDF]

Table S1 Primers used in this article

Primer number	Primers sequence
1	GAACCAGCATTAGTAACACATCATTTTTTTCTCTGTTCTTCACTATTTCTTGA<br>AAAACTAAGAAGTACGCATCAAACTTGGTGAGCGCTAGGAGTCACTGC
2	GCAAATTGATGCTCTTAGAAAATGAAACGTAGTGTTTATGAAGGGCAGGGG<br>GGAAAGTAAAAAACTATGTCTTCCTAGTATCATACTGTTCGTATACATAC
3	GAAAGTAAAAAACTATGTCTTCCT
4	AGTATCATACTGTTCGTATACATAC
5	CCGAGCTCAAAACAATGTTACTAATCATATCATTCACC
6	ACCGCTCGAGCTAACTCCTTTCTTGGATCATAA
7	CGGGGTACCAAAACAATGACTTCTGCTTTGTATGCTTCCG
8	CCGGAATTCTCACCAGACATCTCTGAGGTATC
9	GGAAGATCTACTAGTACGGATTAGAAGCC
10	ACGCGTCGACCCGATTCATTAATGCAGGGC
11	GGAAGATCTGGATCATCCCCACGCGCCCTGTA
12	ACGCGTCGACCCAACGCGCGGGGAGAGGCGGTTT
13	ACGCGTCGACACGGATTAGAAGCCGCCGA
14	ACGCGTCGACAGCTTGCAAATTAAAGCCTTCG
15	CGGACTAGTGGATCCCGACACCCATGAGTGCACCTGAAGGTTT
16	CCCAAGCTTCTATAATTTAGATTCAGAGACTTG
17	CGGACTAGTGGATCCCGACACCCATGAGTGCTTCCAAAATGGCCA
18	CCCAAGCTTTCAGAACTTGGCTCGAATGTC
19	CGGACTAGTGGATCCCGACACCCATGGCGGACACGTTATTGATT
20	CCCAAGCTTTTATGAGTCATGATTTACTAA
