# Supplementary material for: Designing and Creating a Synthetic Omega Oxidation Pathway in Saccharomyces cerevisiae Enables Production of Medium-Chain α, ω-Dicarboxylic Acids
Source: Front Microbiol. 2017 Nov 7;8:2184. doi: 10.3389/fmicb.2017.02184 (PMC5673993; doi:10.3389/fmicb.2017.02184)
Supplement: Supplementary file 2 [file Image_1.pdf]

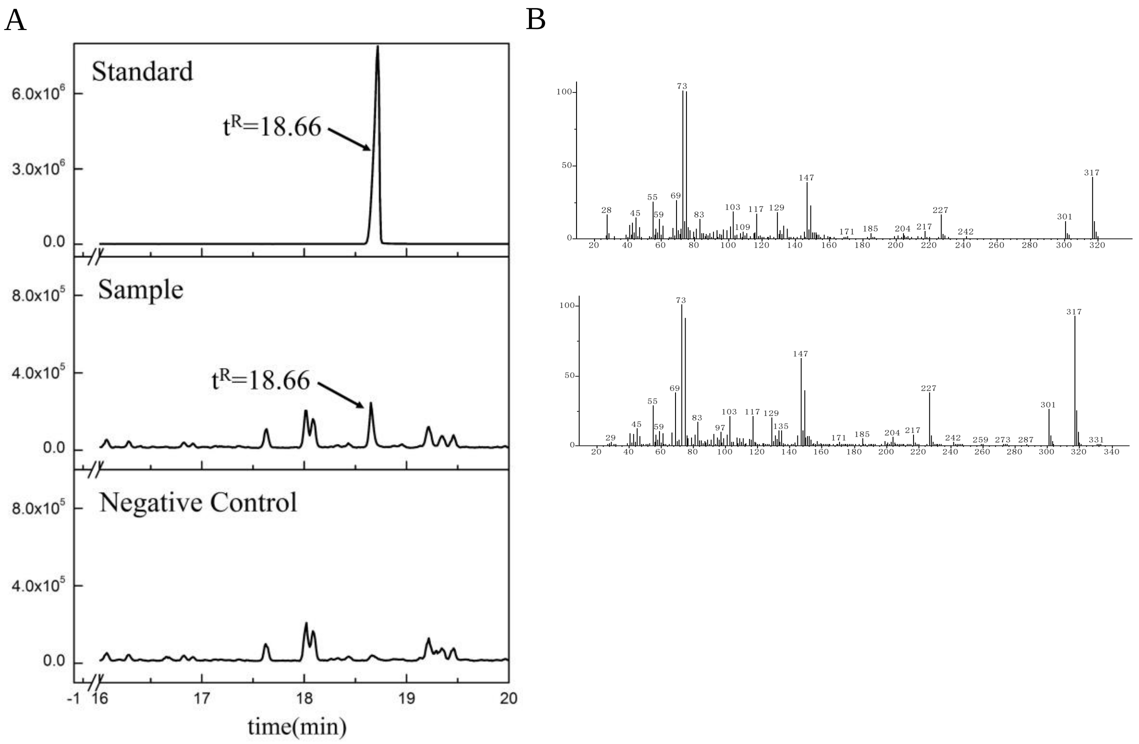

Figure S1 *in vitro* enzyme characterization of *S. cerevisiae* expressing *CYP94C1* and *ATR1*. **(A)** GC-MS Profile of standard 10-hydroxydecanoic acid and microsome of BY4741 expressing *CYP94C1* and *ATR1* with 100 μM substrate decanoic acid. Negative control was BY4741 with empty plasmid pYES2/CT without CYP94C1. **(B)** Ion spectra of EI mass of retention time 18.66 of standard and sample of microsome of BY4741 coexpressing *CYP94C1* and *ATR1* with 100 μM decanoic acid.
