# Supplementary material for: Designing and Creating a Synthetic Omega Oxidation Pathway in Saccharomyces cerevisiae Enables Production of Medium-Chain α, ω-Dicarboxylic Acids
Source: Front Microbiol. 2017 Nov 7;8:2184. doi: 10.3389/fmicb.2017.02184 (PMC5673993; doi:10.3389/fmicb.2017.02184)
Supplement: Supplementary file 3 [file Image_2.pdf]

A

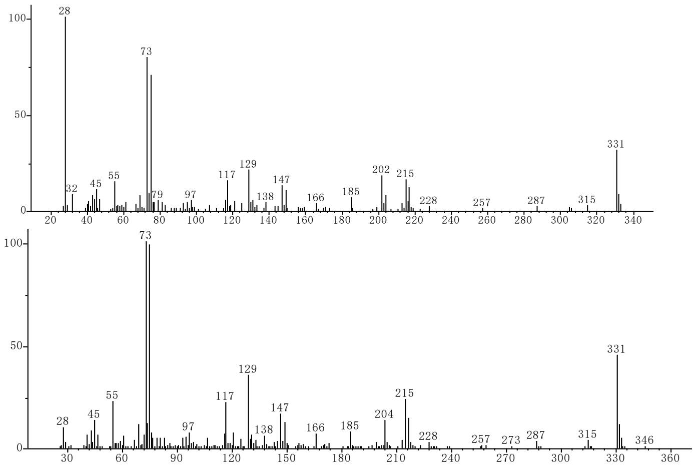
100
50
0
28
73
32
45
55
79
97
117
129
138
147
166
185
202
215
228
257
287
315
331
20 40 60 80 100 120 140 160 180 200 220 240 260 280 300 320 340
100
50
0
73
28
45
55
97
117
129
138
147
166
185
204
215
228
257
273
287
315
331
346
30 60 90 120 150 180 210 240 270 300 330 360

B

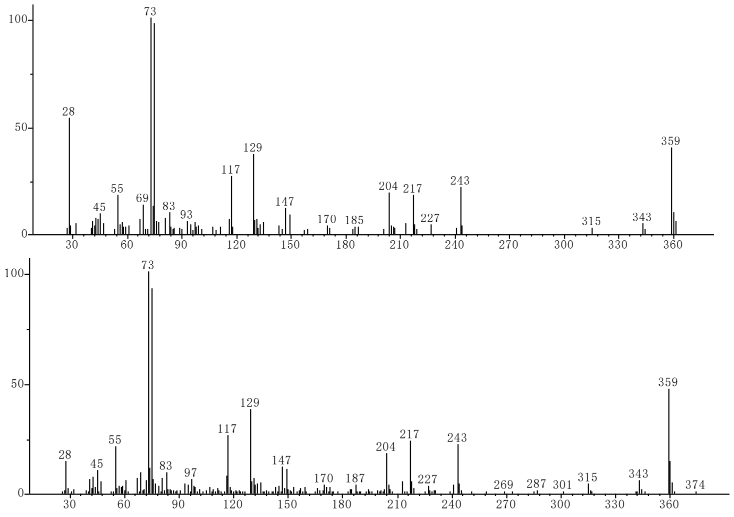
100
50
0
73
28
45
55
69
83
93
117
129
147
170
185
204
217
227
243
315
343
359
30 60 90 120 150 180 210 240 270 300 330 360
100
50
0
73
28
45
55
83
97
117
129
147
170
187
204
217
227
243
269
287
301
315
343
359
374
30 60 90 120 150 180 210 240 270 300 330 360

C

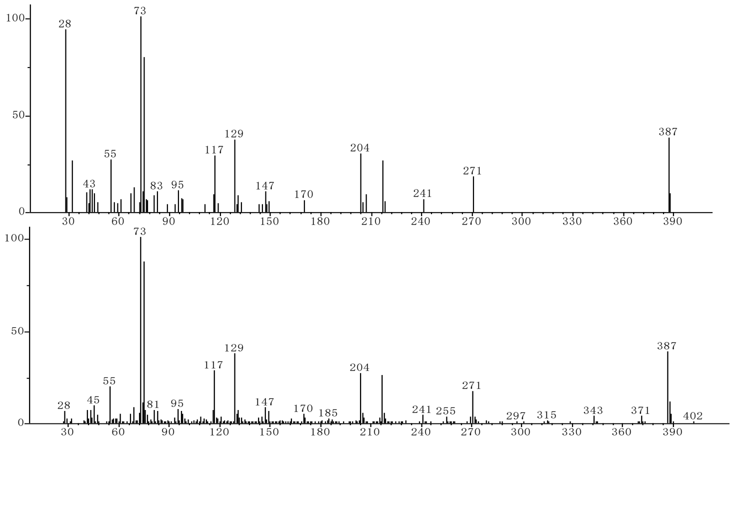
100
50
0
28
73
43
55
83
95
117
129
147
170
204
241
271
387
30 60 90 120 150 180 210 240 270 300 330 360 390
100
50
0
73
28
45
55
81
95
117
129
147
170
185
204
241
255
271
297
315
343
371
387
402
30 60 90 120 150 180 210 240 270 300 330 360 390

D

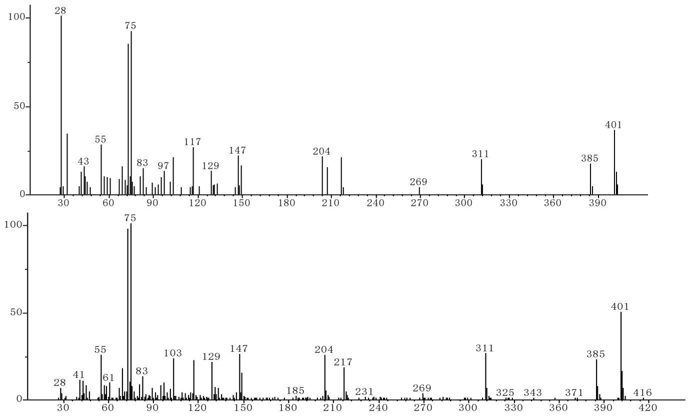

E

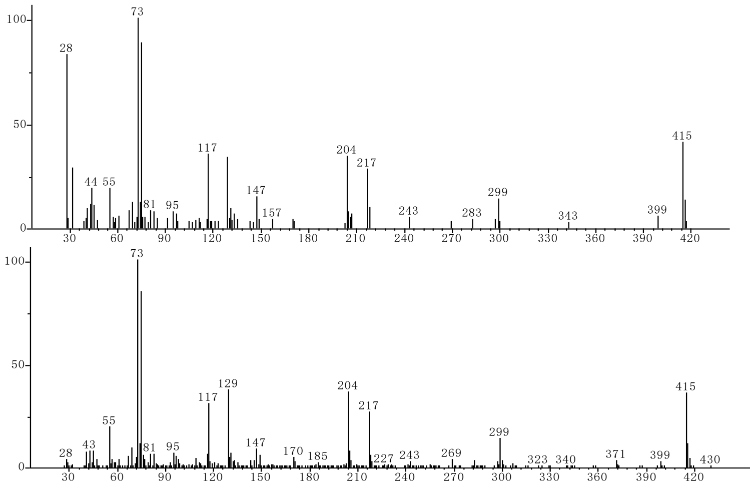

Figure S2 GC-MS confirmation of the production of dicarboxylic acids with engineered strains compared with standards. (a) decanedioic acid; (b) dodecanedioic acid; (c) tetradecanedioic acid; (d) 16-hydroxyhexadecanoic acid; (e) hexadecanedioic acid were verified by comparing the mass spectrum of the analyte (above) against the reference standards (below).
