# Supplementary material for: Designing and Creating a Synthetic Omega Oxidation Pathway in Saccharomyces cerevisiae Enables Production of Medium-Chain α, ω-Dicarboxylic Acids
Source: Front Microbiol. 2017 Nov 7;8:2184. doi: 10.3389/fmicb.2017.02184 (PMC5673993; doi:10.3389/fmicb.2017.02184)
Supplement: Supplementary file 4 [file Image_3.pdf]

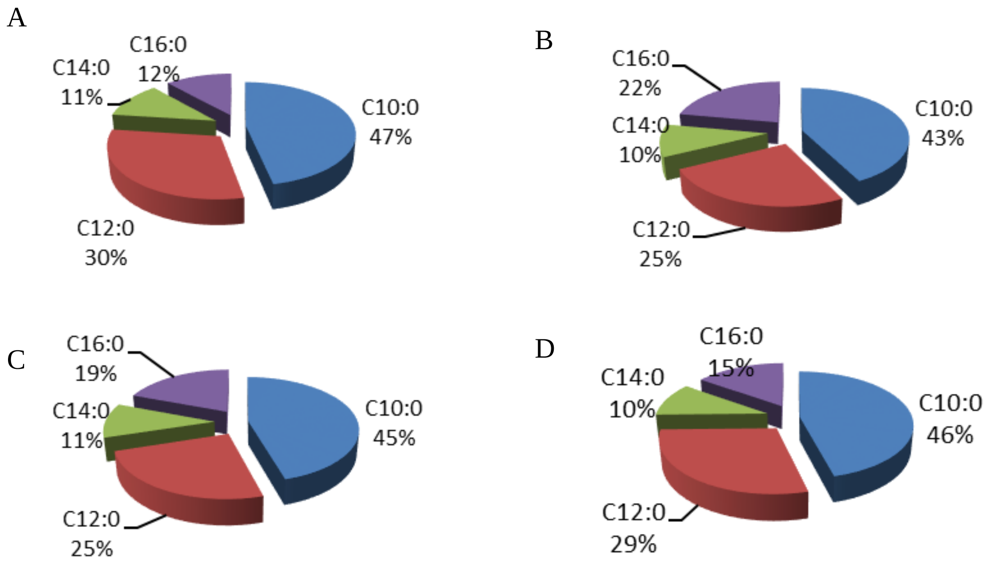

Figure S3 The ratio of dicarboxylic acids of engineered *S.cerevisiae* overexpressing thioesterase gene. **(A)** *Acot8*; **(B)** *PTE1* ; **(C)** *'TesA*; **(D)** *UcfatB*.
