# Supplementary material for: Designing and Creating a Synthetic Omega Oxidation Pathway in Saccharomyces cerevisiae Enables Production of Medium-Chain α, ω-Dicarboxylic Acids
Source: Front Microbiol. 2017 Nov 7;8:2184. doi: 10.3389/fmicb.2017.02184 (PMC5673993; doi:10.3389/fmicb.2017.02184)
Supplement: Supplementary file 5 [file Image_4.pdf]

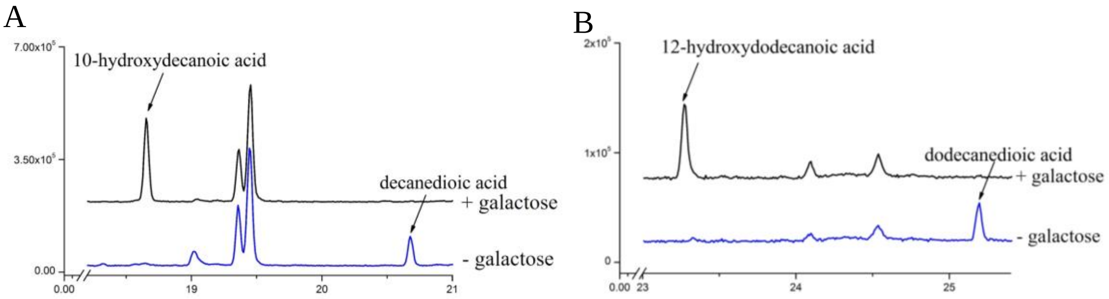

Figure S4 Conversion characteristics of decanoic acid (A) and dodecanoic acid (B) with engineered *S.cerevisiae* expressing *CYP94C1* and *ATR1* with and without galactose after 24h incubation.
