# Supplementary material for: Designing and Creating a Synthetic Omega Oxidation Pathway in Saccharomyces cerevisiae Enables Production of Medium-Chain α, ω-Dicarboxylic Acids
Source: Front Microbiol. 2017 Nov 7;8:2184. doi: 10.3389/fmicb.2017.02184 (PMC5673993; doi:10.3389/fmicb.2017.02184)
Supplement: Supplementary file 6 [file Image_5.pdf]

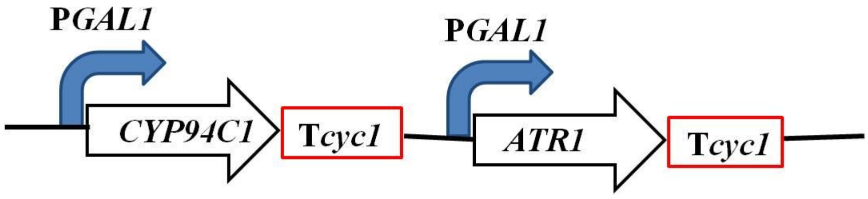

Figure S5 Schematic diagram of the expression cassette for cytochrome P450 gene *CYP94C1* and cytochrome reductase gene *ATR1*.
